# (Cost)-effectiveness of case-management by district nurses among primary informal caregivers of older adults with dementia symptoms and the older adults who receive informal care: design of a randomized controlled trial [ISCRTN83135728]

**DOI:** 10.1186/1471-2458-5-133

**Published:** 2005-12-12

**Authors:** Aaltje PD Jansen, Hein PJ van Hout, Harm WJ van Marwijk, Giel Nijpels, Martine C de Bruijne, Judith E Bosmans, Anne-Margriet Pot, Wim AB Stalman

**Affiliations:** 1 Institute for Research in Extramural Medicine, VU University medical center, Van der Boechorststraat 7, 1081 HV Amsterdam, the Netherlands; 2 Department of General Practice, VU University medical center, Van der Boechorststraat 7, 1081 HV Amsterdam, The Netherlands; 3 Health Technology Assessment Unit, VU University medical center, Van der Boechorststraat 7, 1081 HV Amsterdam, The Netherlands; 4 Department of Nursing Home Medicine, VU University medical center, Van der Boechorststraat 7, 1081 HV Amsterdam, The Netherlands; 5 Trimbos-institute, Netherlands Institute of Mental Health and Addiction, P. O. 725, 3500 AS Utrecht, the Netherlands

## Abstract

**Background:**

Dementia is an incurable disease with devastating consequences for both patients and their relatives. The objective of this study is to describe the study protocol of a randomized controlled trial with assignment to either usual care or case-management by district nurses, among informal caregivers of older adults with dementia symptoms who live at home and the older adults who receive informal care.

**Methods/design:**

In this randomized controlled trial, effectiveness as well as cost-effectiveness of case-management is evaluated. It concerns case-management in early-detected patients with dementia symptoms and their primary informal caregivers. Participants are followed up to twelve months after baseline assessment. The main outcome measure of the effect evaluation is the caregiver's sense of competence to care for the older person with dementia symptoms. The economic evaluation is performed from a societal perspective.

**Discussion:**

This is one of the first trials on case-management that includes an economic evaluation. In addition, it concerns a tailor-made intervention in early-detected patients with dementia symptoms and their caregivers. The results of this randomized controlled trial will provide valuable information for health professionals and policy makers on effectiveness and cost-effectiveness of early tailor-made case-management for patients and their informal caregivers. Moreover, positive effects will challenge current health care systems to move to more pro-active approaches for this group.

## Background

Dementia is a major public health problem with enormous costs to society [1]. It is an incurable progressive disease with devastating consequences for both patients and their relatives. The estimated prevalence rate of dementia among older adults aged 65 to 95 is 6.6% [2]. Over the next years the number of demented older persons will increase substantially as a result of aging populations [3].

Initially, informal caregivers, such as relatives, neighbors and friends, care for most patients with dementia. Caregiving is generally unplanned and most informal caregivers gradually adopt their role because of the insidious nature of dementia [4]. However, informal caregivers often experience adverse psychological, physical, social, and financial consequences [5]. Compared with non-caregivers, they live shorter and report more depressive symptoms [6,7]. Besides, caring for a demented person is marked by losses of previous roles in a relationship. Moreover, many caregivers reduce or give-up the time spent on paid jobs and social activities [4].

Timely detection of dementia is important for both patients and their caregivers as it enables care support and prepares future care [8]. However, there is evidence of underdetection [9,10] and diagnostic delay [11]. An important patient related barrier to timely recognition is the absence of a request for help. This absence can be attributed to denial, labeling cognitive impairment as an accepted aspect of normal ageing, lack of awareness of the disease process, or the idea that nothing can be done [12,13]. In contrast to conventional care, pro-active care with timely detection followed by structured care focusing on both demented patients and informal caregivers, may be more suitable for this vulnerable group. So far, randomized controlled trials of such pro-active disease management systems have not been reported. Yet, up till now, to assist informal caregivers of demented older adults, several psychosocial support programs have been developed, such as support groups, respite care, stress-management, social skills training, psycho-educational groups, and case-management. On the whole, multicomponent interventions that provide caregivers with diverse services and supports, and individually tailored interventions showed larger effects on caregivers' well-being than other, narrowly focused interventions [14-18]. We use the concept 'sense of competence' to denote the caregiver's feeling of being capable to care for the demented person.

Interventions showed increased caregivers' sense of competence [19,20], stabilized caregivers' well-being [21,22] to sustained benefit in reducing depressive symptoms [23], changed caregiver's appraisals of patient behavioral problems [24], and, lastly, postponement of patients' institutionalization [19,20,25-28], although there is lack of strong findings in general [18,29,30]. Trials on case-management, showed a deferral or no reduction in patients' institutionalization rate [27,31], and on the whole did not impact caregivers' levels of depression and burden, in spite of small reductions at some sites [21]. Few studies have performed economic evaluations of interventions for community-dwelling dementia patients [32-34]. Cost-effectiveness analyses and cost-utility analyses are even rare [35].

An innovative initiative to support dementia patients and their caregivers was set up by the Department of General Practice of the VU University medical center, GPs and district nurses in West-Friesland, the Netherlands. We developed a pro-active program, in which the key elements consist of timely detection of dementia symptoms followed by case-management by district nurses among detected patients and their primary informal caregivers. A randomized controlled trial (RCT) is performed to observe effectiveness and cost-effectiveness of case-management.

The objective of this paper was to describe the study protocol of this RCT among informal caregivers of men and women aged 65 years or over with dementia symptoms who live at home, and the men and women they take care of. The main research questions of this RCT concern whether case-management is more effective than usual care in improving caregiver's sense of competence, and whether case-management is cost-effective compared to usual care when assessed from a societal perspective. A secondary research question is whether case-management is more effective than usual care in improving caregiver's quality of life, caregiver's psychological well-being, caregiver's burden, patient's quality of life, and in decreasing hospital days, days until institutionalization and death of the patients.

## Methods/design

### Design

**Figure 1:**
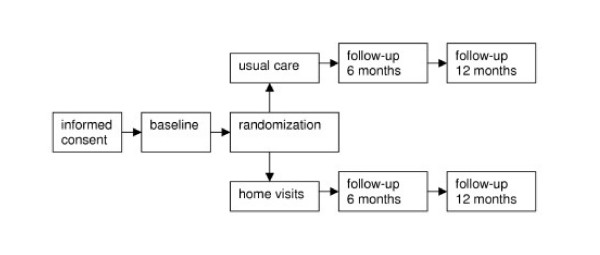
Design

The design is a RCT with assignment to either usual care or case-management by district nurses among patients with dementia symptoms and their primary informal caregivers. Figure 1 shows the design of the study. Participants are only allowed to enter the study after signed informed consent. Representatives give informed consent of incompetent patients. The Medical Ethics Committee of the VU University medical center in Amsterdam approved the study.

### Participants

**Figure 2:**
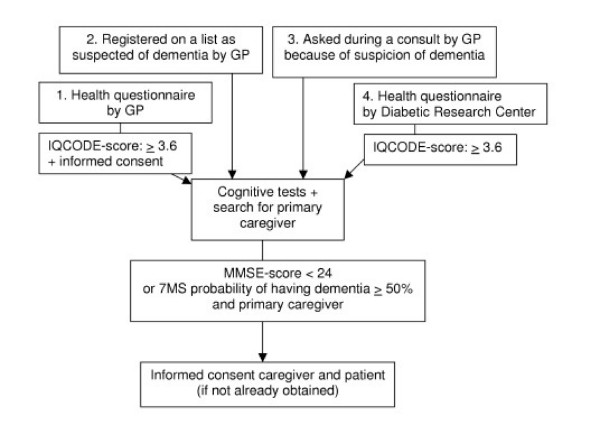
Recruitment of the Study Population

Detection of patients and subsequent recruitment of their informal caregivers takes place among GP patients in West-Friesland, the Netherlands. Patients are potentially eligible for trial entry ifthey are 65 years or over, live outside of institutional settings, and suffer from dementia symptoms. Patients with dementia symptoms have multiple cognitive impairments (i.e. memory impairments, aphasia, apraxia, agnosia, and impairment in executive functioning). These symptoms lead to significant limitations in social functioning and progressive decline in general functioning. Two sources are used to detect patients; 1) Caseload of co-operating GPs. 2) The primary care Diabetic Research Center in which all GPs of West-Friesland participate. Detection of patients takes place in four ways, as shown in Figure 2. 1) GPs who are willing to co-operate, provide a list of addresses of all their patients, aged 75 or over and living at home. All patients receive a postal health questionnaire in order to identify older adults with cognitive decline, as assessed with a self-report version of the short Informant Questionnaire on Cognitive Decline (IQCODE) [36]. 2) Co-operating GPs mark patients who they suspect of dementia on the list of addresses they provide. 3) All GPs in West-Friesland invite patients suspected of dementia after consultation, for a cognitive assessment. 4) The primary care Diabetic Research Center provides addresses of their community-dwelling diabetic patients aged 65 or over and not approached formerly. Older patients with diabetes mellitus are more at risk of dementia and cognitive decline than those with normal glucose tolerance [37]. Diabetic patients also receive an IQCODE.

Patients with an IQCODE score of 3.6 or over (strongly suggesting cognitive decline), and patients suspected of dementia by their GP, are assessed at home with the 7 minute screen (7MS) [38] and the Mini Mental State Examination (MMSE) [39]. Patients who score less than 24 on the MMSE or who have a probability of having dementia of 50% or more according to the 7MS, are considered eligible for trial entry. If an eligible patient has more than one informal caregiver, the primary caregiver is the one who spends most hours on caregiver tasks and who coordinates the caring process. Exclusion criteria for patients applied at baseline are: assistance by an outpatient geriatric team for cognitive problems, terminal illness, insufficient command of the Dutch language, participation in other research projects, and institutionalization. Exclusion criteria for caregivers are: terminal illness and insufficient command of the Dutch language. Recruitment commenced in spring 2003 and ended in summer 2005.

### Randomization

Randomization takes place after baseline measurement. An external independent person establishes the random order using random number tables. Blocking by practice (blocks of four) is used to ensure that comparison groups are of approximately the same size per practice.

### Intervention

During one year, three district nurses who are specialized in geriatric care, act as case-manager of both patient and informal caregiver. Case-management entails assessment, planning, coordination, collaboration, and monitoring of care. Nurses provide practical, informational and socio-emotional support. Multiple support strategies (e.g. support groups, respite care) are offered to informal caregivers and patients. The nurses start the intervention with a home-visit in which they administer a patient assessment; the Resident Assessment Instrument Home Care (RAI-HC). The RAI-HC is a computerized multidimensional instrument that consists of a Minimum Data Set (MDS) that assesses general functioning of the patient, and Client Assessment Protocols (CAPS), providing protocols for the management of 30 potential and actual problem areas [40]. Together with the participants, the nurses order the identified problems of the RAI into a hierarchy, and formulate a care-plan for these problems. Subsequently, they leave behind a form to register care received and appointments with health professionals. In the second home-visit, nurses explore the caregiver's situation with a capacity and burden questionnaire [41] and hand a guide to caregivers holding available social services and welfare professionals. The nurses formulate a care plan for the informal caregiver based on the capacity and burden questionnaire. After these two visits, the nurses and participants decide how they want to proceed with the intervention. When more visits are not necessary, the nurses contact the participants at least every 3 months to monitor their situation. The nurses leave a dossier at the patient's house. This dossier contains the care plan, identified problems by RAI assessment and notes of planned and undertaken activities. Other visiting health professionals may take notice of the dossier and add their own notes. The nurses contact the GPs to inform them about the situation. Apart from these compulsory activities, the intervention holds some tailor-made activities. When necessary, nurses refer to other health professionals, including diagnostic services and monitor the anticipated effect. In addition, the nurses may organize family-meetings to educate relatives, improve social support and relieve the caregiver [4]. Nurses were trained in working with the computerized RAI-HC, and in organizing family-meetings. They also received seminars on how to deal with dementia patients and their patients. They meet monthly to discuss innovations and geriatric cases while supervised by a staff member. Nurses provide care according to a National Guideline on dementia for district nurses [42].

### Usual care

Patients and informal caregivers in the control group receive usual care. In the Netherlands, all people are registered in a primary care practice. General practitioners, as well as a regional indication institution act as gatekeepers of the Dutch health care system. GPs provide cure according to the Guideline on dementia of the Dutch College of General Practitioners. They aim to diagnose and inform dementia patients and their relatives preferably at an early stage [43]. However, guideline recommendations in general, are followed in on average 67% of the decisions [44]. Co-operating GPs are unaware of patients allocated to usual care, unless participants reveal their allocation. Participants of the usual care group have no access to most of the structured and tailor-made activities of the intervention (e.g. family meetings, RAI-HC assessment, guide for informal caregivers). In the region of research, suspected patients are referred to mental health professionals, never to district nurses. All participants of the usual care group are offered the intervention after the one-year follow-up.

### Measurements

Table 1 provides an overview of all effect and economic measurements. At baseline (T0), and after 6 (T1) and 12 months (T2) trained interviewers visit participants. At baseline and after 6 months, they leave cost diaries for patients and caregivers to register medical consumption during the successive 6 months. These cost diaries also provide the possibility to visualize delivery of the intervention and usual care. When patients are unable to fill out a questionnaire, their informal caregiver is allowed to fill it out or to provide assistance.

**Table 1:** Measurement Scheme

**variable**	**Instrument**	**T0**	**T1**	**T2**
**Effect evaluation: primary outcome**				
a. Sense of competence	SCQ [45]	X	X	X
**Effect evaluation: secondary outcomes**				
b. Quality of life of the caregiver	SF-36 [48]	X	X	X
	EQ 5-D [57]	X	X	X
c. Psychological well-being of the caregiver	CES-D [49]	X	X	X
d. Caregiver's burden	SPPIC [50]	X	X	X
e. Days until institutionalization of the patient	GP	continuous registration		
f. Quality of life of the patient	DQOL [51]	X	X	X
	EQ 5-D [57]	X	X	X
g. Days until death of the patient	GP	continuous registration		
h. Hospital days of the patient	Cost diaries	continuous registration		
**Economic evaluation**				
i. Direct and indirect costs	Cost diaries and home-care organisation	continuous registration		

#### Effect evaluation

Primary outcome is:

1. Caregiver's sense of competence as measured with the Sense of Competence Questionnaire (SCQ) [45].

The SCQ consists of three domains, identified by factor analysis: consequences of involvement in care for the personal life of the caregiver, satisfaction with one's own performance as a caregiver and satisfaction with the impaired person as a recipient of care. The questionnaire was based on Zarit's Burden Inventory [46] and Bengtson and Kuypers' family crisis model [47].

Secondary outcomes are:

2. Caregiver's quality of life by means of the MOS 36-item short-form health survey (SF-36) [48];

3. Caregiver's psychological well-being as determined with the Center for Epidemiologic Studies Depression Scale (CES-D) [49];

4. Caregiver's burden by means of the Self-Perceived Pressure by Informal Care (SPPIC) [50];

5. Days until institutionalization of the patient as checked with GP records;

6. Patient's quality of life as measured with the Dementia Quality of Life Instrument (DQOL) [51];

7. Days until death of the patient as checked with GP records;

8. Hospital days of the patient by means of cost diaries.

Apart from these outcomes, we assess the following potential confounding variables on the level of the caregiver: socio-demographic characteristics, disabilities in activities of daily living (ADL) functioning and instrumental activities of daily living (IADL) functioning by means of the Groningen Activity Restriction Scale (GARS) [52], presence of chronic diseases, locus of control (Mastery) [53], and social support (social support list) [54]. On patient level we assess socio-demographic characteristics, cognitive functioning (MMSE, 7MS, IQCODE), presence of chronic diseases, ADL and IADL disability with the Interview for Deterioration in Daily living activities in Dementia (IDDD) [55], behavioral problems and mood by means of the Neuropsychiatric Inventory (NPI-Q) [56], and incontinence.

#### Economic evaluation

The economic evaluation is performed from a societal perspective. The evaluation is a combination of a cost-effectiveness analysis on caregiver's sense of competence (SCQ) and two cost-utility analyses on caregivers and patients separately. Utilities are based on the EQ-5D [57]. Quality Adjusted Life Years (QALY) are calculated by multiplying the utility with the amount of time a patient spends in this particular health state [58]. Incremental costs per QALY gained are calculated. In all analyses, direct costs inside and outside health care are considered. Besides, indirect costs of productivity loss of caregivers and indirect costs of the intervention are estimated. Direct costs inside health care (e.g. costs of consulting the GP, hospitalizations, and use of medication), direct costs outside health care (e.g. costs of traveling, costs of informal care, and costs of consulting alternative health professionals), and productivity loss are assessed by means of cost diaries for caregivers and patients, in which subjects register the amount of healthcare they use. Indirect costs of the intervention, such as nurses' trainings, are calculated using the bottom-up method, by measuring all resources and multiplying these by associated cost prices. Dutch guidelines for economic evaluations in health care are followed to estimate costs [59].

### Sample size calculation

Sample size calculations were based on scores reported for groups similarly to our target group on the main outcome measure of the RCT, namely sense of competence (mean 17.9 SD 5.2, range 4–27) [60]. Calculations are based on α = 0.05 and a desired power of 0.80. For an anticipated effect of 15% difference in final scores between intervention group and usual care group, and with improved scores in the intervention group and stable scores in the control group, 37 persons per group are required. As we expect a drop out rate of about 25% during the one-year follow-up, this means a total of 100 patients and caregivers to be included in the study.

### Blinding

Interviewers are kept blind from the randomization status of participants. GPs can be unaware of patients allocated to the usual care group, but they will be aware of patients in the intervention group as nurses contact the GP about these patients. Participants are not blinded.

### Analysis

#### Effect evaluation

Data are primary analyzed according to the intention-to-treat principle. Additionally, data are analyzed according to the on-treatment (i.e. per protocol) principle in order to examine whether protocol deviations have caused bias. General Linear Models are used to analyze differences between the intervention and usual care group on caregiver's sense of competence, caregiver's quality of life and caregiver's psychological well-being. Potential baseline differences are accounted for by covariates. Differences in days until institutionalization and death between patients of the two groups are tested with survival analyses (Cox-proportional hazard modeling). Differences on patient's hospitalization days and patient's quality of life are tested by a chi-square test and student t-test, respectively. Potential confounding is checked, including the effect of different interviewers and nurses.

#### Economic evaluation

The economic evaluation involves calculating cost-effectiveness and cost-utility ratios. In the pair wise comparison of the mean groups, bootstrapping is used to calculate confidence intervals around the mean difference in costs and ratios. Incremental costs and benefits of the intervention compared to usual care are presented in cost-effectiveness planes and acceptability curves. Substitution of costs is analyzed by describing volumes of healthcare use and associated costs in both groups.

## Discussion

In this paper we described the study protocol of an innovative RCT that evaluates case-management by district nurses to primary informal caregivers of men and woman aged 65 or over with dementia symptoms who live at home, and the older men and women who receive informal care. This is one of the first trials on case-management that includes an economic evaluation. Moreover, it concerns a tailor-made intervention in early-detected patients with dementia symptoms and their caregivers. In addition, the detection method of patients with dementia symptoms preceding recruitment of these patients and their informal caregivers is unique. A large general practice population of older patients was approached by mailed questionnaires. Particular strengths of our study protocol are the randomization approach, in which allocation concealment involves an external independent person, and methods used to enhance the quality of measurements such as assessors who are blinded to group assignment and training of assessors. Another strength is the possibility to visualize delivery of the intervention and usual care by cost diaries. Cost diaries might also provide insight in factors related to the intervention process that may influence the effectiveness of case-management.

Below, we describe design features that address potential threats to reliability and validity. Firstly, selection of participants may limit generalization of the results of this study as selective non-response of older adults, selective refusal of caregivers, and selective dropout are possible. Non-responding older adults in other studies have been observed to have higher rates of functional and cognitive impairment [61,62]. To limit this potential selection bias we will send personalized invitation letters by GPs and provide reminders to initial non-responders. This strategy has shown to be effective [63]. Furthermore, we anticipate that caregivers check mail of cognitively impaired individuals and provide help with filling out as inhabitants are informed about the project by a newspaper article. Selective refusal of caregivers to participate might be assumed as some caregivers will label cognitive impairment as an accepted aspect of normal ageing, or do not experience adverse consequences of caregiving. Possibly, such caregivers will refuse more often than other caregivers. The same might be assumed about severely burdened caregivers who could be afraid to become even more burdened with participating in the project's measurements. To limit such selective refusal, interviewers will contact potential participating caregivers after screening to inform them about the project before sending personalized invitation letters to them. To prevent selective drop-out of severely burdened caregivers and severely disabled patients, appointments for measurements are made by one fixed interviewer on times and locations suitable for the participants.

Secondly, two situations may cause information bias. Firstly, bias may occur as cognitively impaired subjects without insight may fill out questionnaires. However, as we assume that detected patients suffer mainly from mild or moderate dementia symptoms, and insight is mainly preserved in these subjects, this bias probably will be limited. Secondly, in the economic evaluation, caregivers are allowed to provide assistance or to fill out the EQ-5D when patients are unable to fill out this questionnaire. This may lead to information bias, as it is known that agreement on the EQ-5D between patients and caregivers is poor [64]. However, this bias probably will be limited as well, as we assume that detected patients suffer mainly from mild or moderate dementia symptoms, and most patients will fill out the questionnaire themselves.

Thirdly, contamination could bias results of this study as we choose to perform randomization on patient level. However, influence of contamination on results is unlikely as participants of the usual care group have no access to particular activities of the intervention (e.g. family meetings, RAI-HC assessment, guide for informal caregivers). Nevertheless, it is possible that participating GPs are encouraged by the project to give more attention to patients with dementia symptoms and their informal caregivers participating in the usual care group.

Lastly, we expect heterogeneity in study subjects because response to interventions may be different depending on caregiver circumstances. In combination with the relatively small sample size of approximately 100 participants, this heterogeneity may make it hard to interpret the outcomes. However, increasing the sample size is not feasible. Therefore, we will visualize distribution of characterizes over comparison groups to estimate the influence of this heterogeneity on outcome measures. Moreover, cost diaries will detect heterogeneity in received care within the usual care group as well as in the case-management group.

The results of this RCT will provide valuable information for health professionals and policy makers on effectiveness and cost-effectiveness of timely tailor-made case-management for patients and their informal caregivers. Moreover, positive effects will challenge current health care systems to move to more pro-active approaches for this group. In case of proven effectiveness and cost-effectiveness, we recommend implementing this case-management intervention into usual healthcare. The results of this study will be available in autumn 2006.

## Competing interests

The author(s) declare that they have no competing interests.

## Authors' contributions

All authors have read and approved the final version of the manuscript.

AJ is the principal investigator and writer of this manuscript

HH wrote the study protocol and supervises the planning and project

HM wrote the study protocol and supervises the planning and project

GN supervises the planning and project

MB supervises the economic evaluation

JB performs the economic evaluation

AP trained nurses and assisted in putting together the care-program

WS supervises the project

## Pre-publication history

The pre-publication history for this paper can be accessed here:


